# Regenerative Surgical Treatment of Peri-Implantitis: A Systematic Review and Meta-Analysis

**DOI:** 10.3390/dj14030180

**Published:** 2026-03-18

**Authors:** Gabor Fuerst, Shko Atta Ali, Xiaohui Rausch-Fan, Markus Laky

**Affiliations:** 1 Division of Periodontology, University Clinic of Dentistry, Medical University of Vienna, 1090 Vienna, Austria; gabor.fuerst@meduniwien.ac.at (G.F.);; 2 Independent Researcher, 1160 Vienna, Austria; shkoata@gmail.com

**Keywords:** peri-implantitis, regenerative surgery, bone grafts, guided bone regeneration, platelet-rich fibrin, hyaluronic acid, open flap debridement

## Abstract

**Background/Objectives:** This systematic review and meta-analysis evaluated the clinical effectiveness of regenerative surgical treatments compared with open flap debridement (OFD) in the management of peri-implantitis and, secondarily, assessed whether more advanced regenerative approaches, including guided bone regeneration (GBR), platelet-rich fibrin (PRF), and hyaluronic acid (HA), provide additional clinical benefit compared with bone grafting alone. **Methods:** A comprehensive search of PubMed, Scopus, and Web of Science was conducted in accordance with PRISMA guidelines and the PICO model, covering the period from 1993 to 2024. From 2119 identified articles, 63 full-text papers were reviewed, and 12 studies met all inclusion criteria. These studies compared regenerative treatments with OFD and bone grafting using clinical outcomes of probing pocket depth (PPD), radiographic bone level (RBL), bleeding on probing (BOP), suppuration (SUP), mucosal recession (REC), and clinical attachment level (CAL). Meta-analysis was performed using a random-effects model. **Results:** Regenerative treatments demonstrated superior outcomes in radiographic bone level gain compared with OFD (*p* < 0.001), while no statistically significant differences were observed for PPD (*p* = 0.77), BOP (*p* = 0.13), SUP (*p* = 0.42), REC (*p* = 0.14), or CAL (*p* = 0.96). Comparisons between bone grafting and other regenerative materials also showed no statistically significant differences. **Conclusions:** Regenerative procedures improved radiographic bone outcomes but did not consistently outperform OFD in soft tissue parameters, and no advanced regenerative modality demonstrated clear clinical superiority over bone grafting alone. Further high-quality randomized controlled trials with standardized methodologies are needed to establish clinical guidelines for peri-implantitis surgery.

## 1. Introduction

Osseointegrated dental implants are a treatment option that is becoming increasingly common for replacing lost or missing teeth. They support different restorations and have high long-term survival rates (≥10 years). Despite their functionality, implants and their restorations can encounter mechanical and biological complications, meaning that long-term success may not match survival rates [1]. Peri-implant tissues, which surround osseointegrated implants, are classified into soft and hard tissues. The soft tissue, or “peri-implant mucosa,” develops during the wound-healing phase after implant or abutment placement. Hard peri-implant tissue (bone) provides primary stability through direct contact with the implant surface. These tissues serve two fundamental purposes: the bone supports the implant, and the mucosa shields the underlying bone [2].

A review by Araujo and Lindhe concluded that for peri-implant tissues to be considered healthy, there should be no clinical signs of inflammation, including erythema, swelling, or bleeding on probing. The survival and success of the implant may be in danger if the peri-implant tissues are inflamed and destroyed. Peri-implant diseases are categorized into two main types: peri-implant mucositis and peri-implantitis [2].

Peri-implant mucositis is an inflammatory condition affecting the soft tissues around an osseointegrated implant, without loss of supporting bone. Between 39.4% and 80% of dental implant patients have peri-implant mucositis [3]. Radiation, smoking, and biofilm buildup have all been found to be risk factors. Other potential factors such as diabetes, a lack of keratinized mucosa, and the presence of extra luting cement need further investigation [4]. This condition is reversible, and its treatment is the only way to prevent the progression to peri-implantitis [3]. Treatment is similar to non-surgical peri-implantitis therapy, involving mechanical cleaning using curettes, ultrasonic devices, air-abrasive tools, or lasers, with or without local antibiotics or antiseptics. These methods have shown improved clinical metrics such as bleeding on probing in controlled studies [5].

Peri-implantitis is a plaque-associated pathological condition affecting the tissues surrounding dental implants, characterized by inflammation and progressive loss of supporting bone [6]. It presents with bleeding on probing (BOP), suppuration, increased probing depths, mucosal margin recession, and radiographic bone loss [7]. According to a systematic review by Diaz P et al., the prevalence of peri-implantitis varies: 19.53% at the patient level and 12.53% at the implant level [8]. Risk factors include prior periodontitis, poor plaque control, and lack of maintenance [6].

Treatment modalities for peri-implantitis are based on the severity of disease and type of defect. Non-surgical treatment includes mechanical debridement, lasers, chemicals, and antibiotics [9,10]. However, because of the complexity of implant surfaces, non-surgical therapy often leads to only modest improvement [11,12].

Surgical treatment, such as open flap debridement or access flap surgery, enables direct cleaning and decontamination of implant surfaces [5,13,14]. Regenerative surgery, which includes the use of bone grafts, membranes, platelet-rich fibrin (PRF), and hyaluronic acid (HA), aims to restore lost peri-implant bone and improve long-term stability [13,15,16,17,18,19,20,21]. Studies suggest that regenerative approaches can provide better radiographic bone fill compared to OFD alone, though outcomes remain variable and technique-sensitive [18].

Given the lack of consensus on the most effective regenerative strategy and material, this systematic review and meta-analysis was conducted to compare regenerative surgical treatment with open flap debridement and to assess the clinical performance of different regenerative materials. Therefore, the primary aim of this systematic review and meta-analysis was to compare regenerative surgical treatments with open flap debridement (OFD) in the management of peri-implantitis using commonly reported clinical and radiographic outcome measures. In addition, a secondary analysis was performed to assess whether more advanced regenerative approaches, including guided bone regeneration (GBR), platelet-rich fibrin (PRF), and hyaluronic acid (HA), provide any additional clinical benefit compared with bone grafting alone.

## 2. Materials and Methods

### 2.1. Protocol and Search Strategy

This systematic review and meta-analysis were conducted in accordance with the PRISMA (Preferred Reporting Items for Systematic Reviews and Meta-Analyses) guidelines [22]. The research question was formulated using the PICO framework. The population included patients with peri-implantitis. The intervention focused on surgical regenerative treatments, while the comparison groups included open flap debridement (OFD), bone grafting (BG), guided bone regeneration (GBR), platelet-rich fibrin (PRF), and hyaluronic acid (HA). The primary outcomes were reduction in probing pocket depth (PPD), radiographic marginal bone loss (RBL), and bleeding on probing (BOP). Secondary outcomes included suppuration (SUP), recession (REC), and gain in clinical attachment level (CAL). A comprehensive electronic search was carried out in PubMed, Scopus, and Web of Science on 23 November 2024, covering literature published from 1993 to 2024. Specific search strings were used based on the electronic library to identify studies related to peri-implantitis and regenerative surgical treatments involving GBR, PRF, HA, and surgical techniques. The full electronic search strategies for all databases are provided in the Supplementary Materials. The review protocol was not registered in PROSPERO. As this review was based exclusively on previously published studies, ethical approval and informed consent were not required. The PRISMA 2020 checklist is provided as part of the Supplementary Materials.

### 2.2. Selection Criteria

Studies were eligible for inclusion if they investigated regenerative surgical treatments for peri-implantitis and compared at least one of GBR, PRF, or HA to OFD or bone grafting without a membrane. Only human studies published in peer-reviewed journals with a test and a control group were considered. Eligible studies had to report at least one clinical outcome related to PPD, RBL, BOP, SUP, REC, or CAL and be published in English.

Studies were excluded if they were in vitro or animal research, case reports, reviews, editorials, or opinion papers. Additional exclusions applied to studies without control groups, those lacking relevant clinical outcome data, those involving resective surgeries, or articles with insufficient or unclear data.

### 2.3. Data Extraction

Data extraction was initially conducted using a standardized Microsoft Word template and then transferred into Excel for further organization and statistical review. Extracted data included author name, year of publication, study design, number of patients and implants, details of test and control interventions, type of surgical treatment, and follow-up duration. Clinical outcomes recorded included PPD, RBL, BOP, SUP, REC, and CAL. Data extraction was performed independently by two reviewers using a standardized template.

In some studies, bleeding on probing (BOP) was substituted with modified bleeding index (mBI). In such cases, the corresponding authors were contacted to obtain the original BOP values. Only Dr. Alberto Monje responded, and his data were included accordingly. In other cases, BOP data could not be retrieved and were therefore excluded.

For the first and main meta-analysis a total of 23 treatment groups, 7 control groups and 16 test groups were extracted from the 12 included studies. Treatment arms not meeting inclusion criteria, such as those involving laser therapy, were excluded to maintain consistency in the definition of regenerative treatment. Risk of bias was assessed independently for each included study using the Cochrane Risk of Bias tool (RoB 2) for randomized controlled trials and the ROBINS-I tool for non-randomized studies [23,24].

### 2.4. Statistical Analysis

Since many studies reported BOP and related outcomes as percentages without providing raw data, standard error (SE) was calculated using binomial distribution. Meta-analysis was performed using a random-effects model to accommodate clinical and methodological heterogeneity among studies. Mean differences were used for continuous outcomes, while proportion-based estimates were applied for dichotomous outcomes. Forest plots illustrated the effect sizes and confidence intervals. Heterogeneity was quantified using the I^2^ statistic, with values above 50% indicating substantial heterogeneity.

All statistical analyses were performed using Stata/MP version 17 (StataCorp LLC, College Station, TX, USA)). The meta-analysis compared regenerative treatments, bone grafting, GBR, PRF, and HA—against OFD. Subgroup analysis was also conducted to evaluate bone grafting alone versus other regenerative modalities.

## 3. Results

### 3.1. Selection of Studies and Characteristics

**Figure 1:**
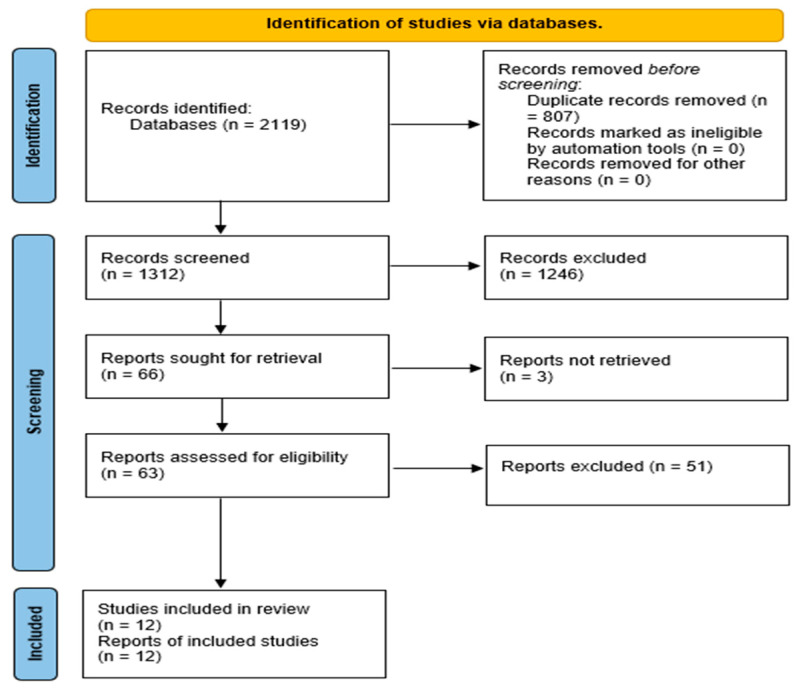
PRISMA 2020 flow diagram illustrating the study selection process.

The selection process followed PRISMA 2020’s approach as can be seen in Figure 1. A total of 2119 studies were identified across the three databases—466 from PubMed, 521 from Scopus, and 1132 from Web of Science. After removing 807 duplicates using EndNote, 1312 articles were retained for title and abstract screening. Based on predefined inclusion and exclusion criteria, 1246 studies were excluded at this stage, and 3 potentially eligible reports could not be retrieved in full text and were therefore excluded from further assessment. Study selection was performed independently by two reviewers, with disagreements resolved through discussion.

Full-text screening was conducted for 63 articles. Of these, 12 studies met all inclusion criteria and were selected for final analysis. These studies included relevant data comparing GBR, PRF, and HA treatments against OFD and/or bone grafting for peri-implantitis (Table 1).

### 3.2. Risk of Bias Assessment

**Figure 2:**
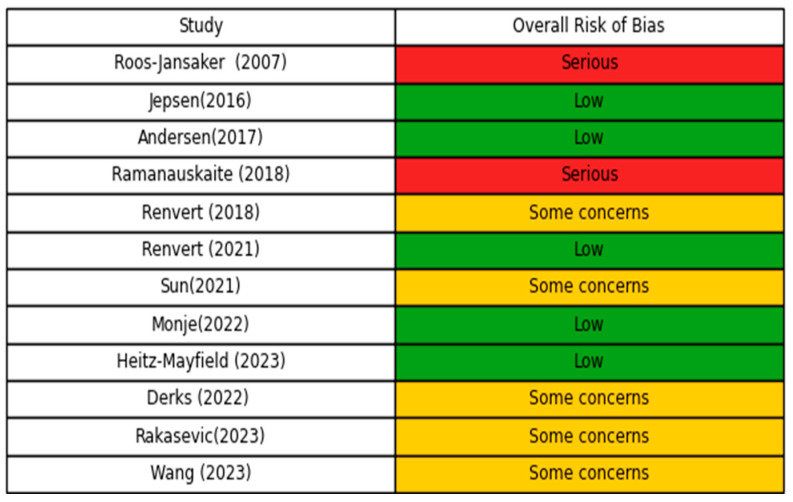
Traffic-light plot of overall risk of bias across included studies (*n* = 12). Roos-Jansaker 2007 [25], Jepsen 2016 [26], Andersen 2017 [27], Ramanauskaite 2018 [28], Renvert 2018 [29], Renvert 2021 [30], Sun 2021 [20], Monje 2022 [31], Heitz-Mayfield 2023 [32], Derks 2022 [33], Rakasevic 2023 [34], Wang 2023 [35].

The overall risk of bias of the included studies is shown in Figure 2. Five studies were assessed as having a low risk of bias, five raised some concerns, and two were judged to have a serious risk of bias.

### 3.3. First Meta-Analysis

#### 3.3.1. Radiographic Bone Level (RBL)

**Figure 3:**
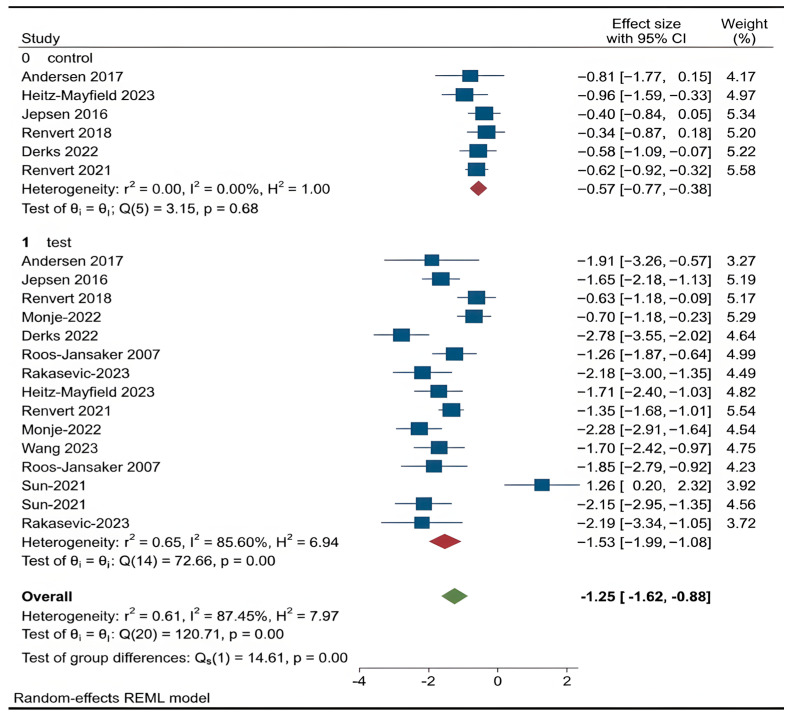
Forest plot for RBL. Roos-Jansaker 2007 [25], Jepsen 2016 [26], Andersen 2017 [27], Renvert 2018 [29], Renvert 2021 [30], Sun 2021 [20], Monje 2022 [31], Heitz-Mayfield 2023 [32], Derks 2022 [33], Rakasevic 2023 [34], Wang 2023 [35].

According to the first meta-analysis, regenerative treatments (bone graft, GBR, PRF, HA) showed a statistically significant improvement over open flap debridement (OFD) only in radiographic bone level gain (RBL) (*p* < 0.001) (Figure 3).

#### 3.3.2. Other Outcomes

For all other clinical outcomes, there were no statistically significant differences observed between the two treatment methods:

PPD (*p* = 0.77),
BOP (*p* = 0.13),
SUP (*p* = 0.42),
REC (*p* = 0.14),
CAL (*p* = 0.96).

**Figure 4:**
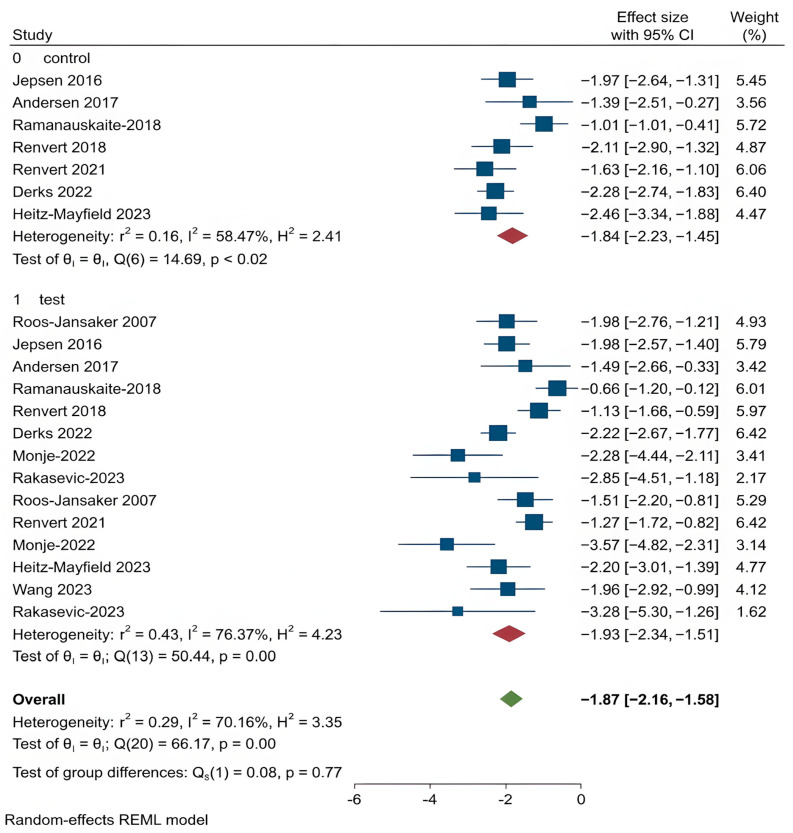
Forest plot for PPD. Roos-Jansaker 2007 [25], Jepsen 2016 [26], Andersen 2017 [27], Ramanauskaite 2018 [28], Renvert 2018 [29], Renvert 2021 [30], Monje 2022 [31], Heitz-Mayfield 2023 [32], Derks 2022 [33], Rakasevic 2023 [34], Wang 2023 [35].

**Figure 5:**
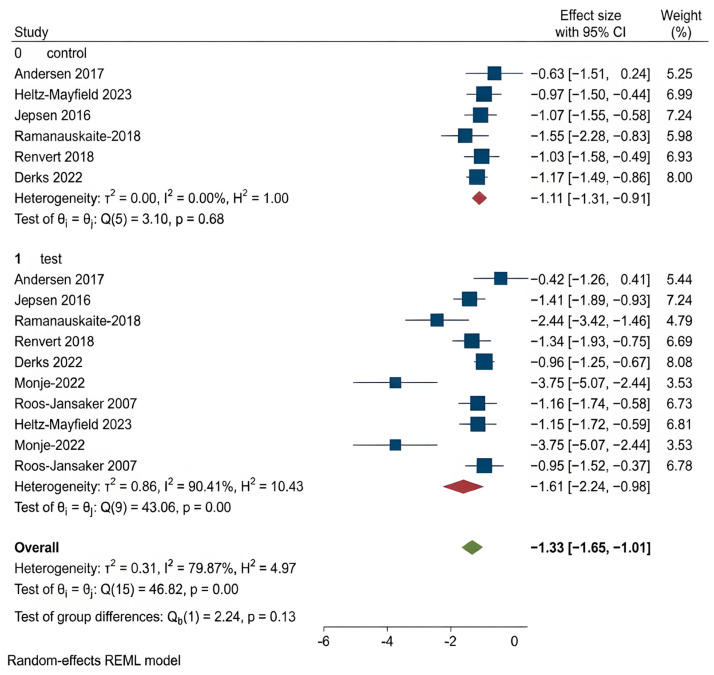
Forest plot for BOP. Roos-Jansaker 2007 [25], Jepsen 2016 [26], Andersen 2017 [27], Ramanauskaite 2018 [28], Renvert 2018 [29], Monje 2022 [31], Heitz-Mayfield 2023 [32], Derks 2022 [33].

**Figure 6:**
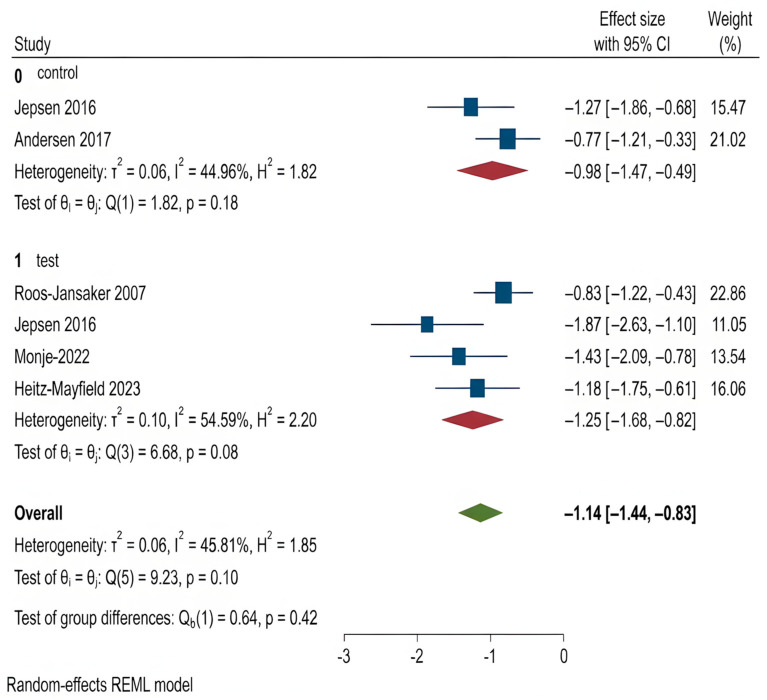
Forest plot for SUP. Roos-Jansaker 2007 [25], Jepsen 2016 [26], Andersen 2017 [27], Monje 2022 [31], Heitz-Mayfield 2023 [32].

**Figure 7:**
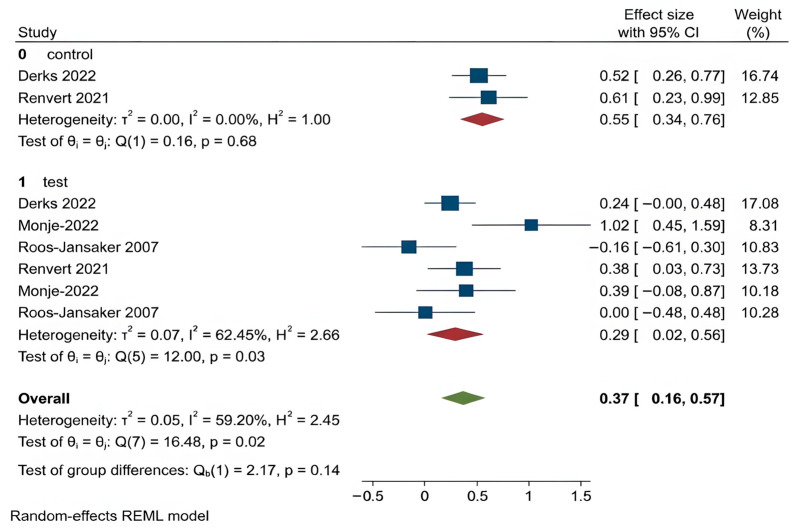
Forest plot for REC. Roos-Jansaker 2007 [25], Renvert 2021 [30], Monje 2022 [31], Derks 2022 [33].

**Figure 8:**
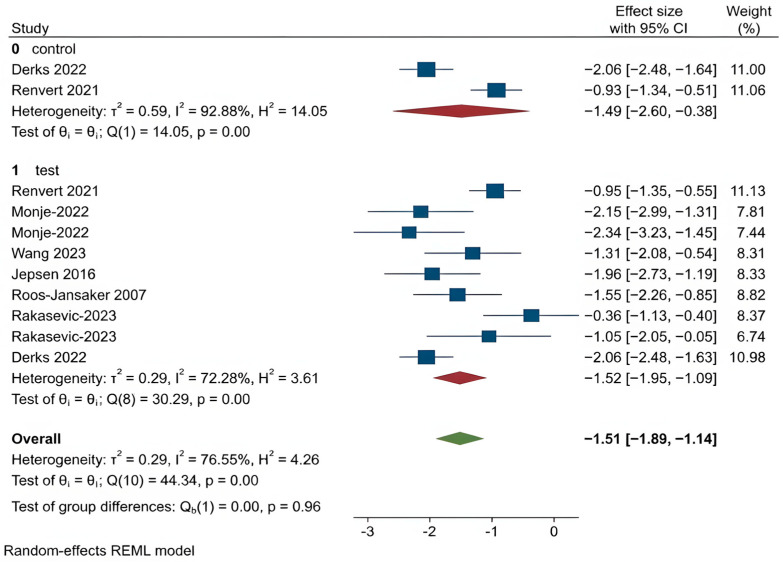
Forest plot for CAL. Roos-Jansaker 2007 [25], Jepsen 2016 [26], Renvert 2021 [30], Monje 2022 [31], Derks 2022 [33], Rakasevic 2023 [34], Wang 2023 [35].

These findings suggest that while regenerative procedures appear more effective in promoting bone regeneration, they do not consistently outperform OFD in improving other clinical parameters related to peri-implantitis. The corresponding forest plots for these outcomes are shown in Figure 4, Figure 5, Figure 6, Figure 7 and Figure 8.

### 3.4. Second Meta-Analysis

The second meta-analysis compared bone grafts to other more advanced regenerative materials (GBR, PRF, HA). None of the clinical outcomes showed statistically significant differences between the treatment groups. While some test groups presented slightly improved numerical results, these differences were not statistically significant. This suggests that, based on the currently available data, no specific regenerative material demonstrates a clear clinical advantage over others in the surgical treatment of peri-implantitis.

## 4. Discussion

This systematic review and meta-analysis assessed the effectiveness of various surgical methods for peri-implantitis treatment through evaluation of six clinical metrics: probing pocket depth (PPD), radiographic bone level (RBL), bleeding on probing (BOP), suppuration (SUP), mucosal recession (REC), and clinical attachment level (CAL). Two independent subgroup analyses were conducted. The first evaluated open flap debridement (OFD) versus regenerative treatments (bone graft, guided bone regeneration (GBR), platelet-rich fibrin (PRF), and hyaluronic acid (HA)). The second compared bone grafts to more advanced regenerative treatments. In the twelve studies included, different regenerative materials were applied for the surgical management of peri-implantitis. The grafting approaches involved xenografts, allografts, and synthetic substitutes, with some reports also examining the use of porous titanium granules. In certain protocols, grafts were combined with resorbable membranes for guided bone regeneration, whereas others relied on grafting alone. Autogenous grafts were occasionally used in conjunction with ridge augmentation or sinus lift procedures. A number of studies also incorporated platelet-rich fibrin, either mixed with graft material or applied as a membrane. Despite these variations in technique, no single material consistently proved superior across studies.

Regenerative treatments demonstrated better results than OFD for RBL in the first analysis, suggesting superior bone restoration. However, no significant differences were found between OFD and regenerative treatments for PPD, BOP, SUP, REC, or CAL. Regenerative techniques improve radiographic bone outcomes, but their impact on inflammation control and soft tissue parameters remains unclear.

In the second analysis, bone grafts were compared to GBR, PRF, and HA. No significant differences emerged regarding evaluated parameters. Some techniques showed better mean results (e.g., CAL gain with GBR/PRF/HA), but none reached statistical significance. Current evidence does not validate one regenerative method over others.

Due to the limited number of studies in some comparisons, findings should be interpreted with caution.

### 4.1. Outcome-Specific Evaluation

Probing Pocket Depth (PPD) refers to the distance from the gingival margin to the base of the pocket around the implant, and is a primary indicator of peri-implant inflammation and treatment response [15,36,37,38]. Both OFD and regenerative groups showed PPD reductions, with greater average reduction in the regenerative group. However, the difference was not statistically significant. The second analysis found no significant differences between regenerative techniques as well.

Radiographic Bone Level (RBL): RBL is a radiographic measure indicating the level of alveolar bone support around implants, serving as a crucial parameter for disease progression and healing [39,40]. In the first analysis, OFD had a pooled effect size of –0.58 mm versus −1.53 mm in the regenerative group, a statistically significant difference (*p* < 0.001). In the second analysis, both bone graft and GBR/PRF/HA showed RBL gains without significant inter-group difference (*p* > 0.05).

Bleeding on Probing (BOP): BOP is a soft tissue inflammation marker that indicates vascular response upon probing [4,41]. Both treatment groups reduced BOP in the first analysis, but not significantly (*p* = 0.135). The second analysis also showed greater BOP reduction in advanced regenerative groups, but again without significance (*p* = 0.72).

Suppuration (SUP): SUP reflects active peri-implant infection, typically assessed through the presence of exudate during probing or palpation [42,43]. The first analysis revealed that OFD and regenerative methods decreased suppuration but failed to show a meaningful statistical difference between the groups (*p* = 0.422). The test group exhibited slightly better suppression of SUP compared to bone graft in the second analysis yet this difference remained insignificant (*p* = 0.35). These findings demonstrate that both surgical methods demonstrate equal effectiveness in managing infection.

Recession (REC): REC represents the apical migration of the soft tissue margin, influencing both esthetics and implant tissue stability [43,44]. Both groups experienced recession, with slightly less progression in the regenerative group (*p* = 0.863). No significant differences were found in the second analysis as well (*p* = 0.86).

Clinical Attachment Level (CAL): CAL measures the vertical distance from a fixed reference point on the implant to the base of the sulcus and reflects peri-implant tissue integration [45]. Both groups showed CAL gain, but without significant differences (first analysis *p* = 0.963; second analysis *p* = 0.58).

Although test groups generally demonstrated better numerical outcomes, statistical significance was not achieved in most comparisons. Regenerative therapies showed notable radiographic improvement, but consistent clinical superiority remains unproven.

### 4.2. Comparison with Existing Literature

Findings of this review align with previous systematic reviews. Daugela et al. (2016) [16] observed improved outcomes with regenerative treatments but could not confirm superiority due to heterogeneity. Solderer and Schmidlin (2020) [18] reported a 57% average bone fill improvement from regenerative treatments, but design differences limited their conclusions. Aljohani et al. (2020) [46] and Wijesundara et al. (2023) [47] echoed these findings, noting general benefits of regenerative treatments without consistent superiority across all clinical measures.

### 4.3. Patient-Centred Outcomes and Surgical Complications

Some studies reported healing related complications, particularly with membrane use. Roos-Jansåker et al. [25] observed 43.8% membrane exposure and higher wound instability in membrane groups. However, Heitz-Mayfield et al. [32], Derks et al. [33], and Renvert et al. (2021) [30] reported similar patient-reported outcomes between regenerative and non-regenerative groups. No major adverse events were reported, but inconsistent reporting across studies limited conclusions.

Future trials should adopt standardized evaluation of adverse events and validated tools to assess patient experience for a more comprehensive understanding of peri-implantitis treatment.

### 4.4. Limitations

This review faced several limitations. The small number of included studies and significant heterogeneity in surgical protocols, defect morphology, and follow-up durations reduced statistical power. Formal assessment of publication bias was not performed due to the limited number of studies available for most outcomes. BOP and SUP data were estimated due to unavailable raw data, introducing uncertainty. Reclassification of some studies was necessary, though consistently applied. Future studies should employ standardized methods, larger samples, and comprehensive reporting to improve comparative analysis.

## 5. Conclusions

This systematic review and meta-analysis evaluated surgical interventions for peri-implantitis across six clinical outcomes: PPD, RBL, BOP, SUP, REC, and CAL. Regenerative treatments showed a statistically significant advantage over OFD only in radiographic bone level gain. No significant differences were found in soft tissue outcomes, and no advanced regenerative technique (GBR, PRF, HA) outperformed bone grafts.

While regenerative methods appear promising for improving bone levels, their overall clinical superiority remains unconfirmed. These findings highlight the need for further high-quality randomized controlled trials with standardized protocols, extended follow-up periods, and comprehensive evaluation of defect morphology, as defect configuration may significantly influence the clinical outcomes of regenerative procedures.

## Figures and Tables

**Table 1:** Summary of extracted data from included studies.

Study	Study Type	Groups	Number of Implants	Outcomes	Outcomes Numbers	Mean or Percent	Standard Deviation	Result
Roos-Jansaker 2007 [25]	PCS	Test group: BG	19	PPD	19	2.22	1.58	Both approaches improved peri-implantitis at 1 year (PD reduction ~3 mm, defect fill ~1.4–1.5 mm, BOP greatly reduced) with no significant difference between membrane and no-membrane groups.
				RBL	19	1.44	1.27	
				BOP	19	25		
				REC	19	1.61	1.61	
				CAL	19	3.83	1.78	
		Test group: GBR	17	PPD	17	2.58	2	
				RBL	17	1.52	1.16	
				BOP	17	22		
				REC	17	1.38	1.5	
				CAL	17	3.96	1.87	
Jepsen 2016 [26]	RCT	Test group: BG	33	PPD	33	3.5	1.5	BG achieved significantly greater radiographic defect fill (*p* < 0.0001) compared with OFD, while clinical improvements (PPD and BOP reduction) were similar between groups.
				RBL	33	1.98	1.99	
				BOP	33	33.3		
				SUP	33	1		
		Control group: OFD	26	PPD	26	3.5	1.1	
				RBL	26	3.63	2.34	
				BOP	26	40.4		
				SUP	26	1.3		
Andersen 2017 [27]	Evaluation of RCT	Test group: BG	6	PPD	6	5.3	1.9	BG vs. OFD showed minimal between-group differences in clinical and radiographic outcomes; statistical testing was not performed due to a small sample size.
				RBL	6	2.7	1.3	
				BOP	6	77		
		Control group: OFD	6	PPD	6	4.5	2.1	
				RBL	6	3.2	1.4	
				BOP	6	83		
Ramanauskaite 2018 [28]	RA	Test group: BG	16	PPD	16	4.94	2.18	BG vs. OFD showed similar reductions in PPD and BOP.
				BOP	16	25.04		
		Control group: OFD	16	PPD	16	4.67	2.35	
				BOP	16	45.12		
Renvert 2018 [29]	RCT	Test group: BG	21	PPD	21	3.9	2.7	BG vs. OFD showed significantly greater radiographic defect fill, BOP and PPD did not differ significantly between groups.
				RBL	21	2.9	1.2	
				BOP	21	52.4		
		Control group: OFD	20	PPD	20	2.6	1.5	
				RBL	20	3.1	1.2	
				BOP	20	65		
Renvert 2021 [30]	RCT	test group: GBR	32	PPD	32	4.8	1.5	GBR vs. OFD showed significantly greater radiographic defect fill, clinical outcomes (PPD, BOP, SUP, REC) and patient-reported outcomes were similar between groups.
				RBL	32	2.1	1.6	
				REC	32	0.8	1.2	
				CAL	32	5.6	1.64	
		Control group: OFD	34	PPD	34	4.5	1.5	
				RBL	34	3.6	2.3	
				REC	34	1.4	1.5	
				CAL	34	5.9	1.73	
Sun 2021 [20]	RCT	Test group: GBR	40	RBL	40	3.97	1.46	PRF + GBR vs. GBR alone showed significantly greater improvement in defect depth/width.
		Test group: PRF	40	RBL	40	1.01	0.18	
Monje 2022 [31]	RCT	Test group: BG	18	PPD	18	3.01	0.72	Both groups showed significant clinical and radiographic improvement; however, no significant differences were found BG and GBR.
				RBL	18	1.73	0.83	
				BOP	18	12.3		
				REC	18	1.54	0.93	
				CAL	18	4.55	1.28	
		Test group: GBR	18	PPD	18	3.13	0.68	
				RBL	18	1.72	0.72	
				BOP	18	12.3		
				REC	18	1.04	0.81	
				CAL	18	4.17	1.22	
				SUP	18	1.4		
Heitz-Mayfield 2023 [32]	RCT	Test group: GBR	20	PPD	20	3.1	1.9	GBR vs. OFD showed no statistically significant differences in clinical or radiographic parameters.
				RBL	20	2.7	1.4	
				SUP	20	30		
				BOP	20	10		
		Control group: OFD	20	PPD	20	3.4	1.8	
				RBL	20	3.6	1.6	
				SUP	20	40		
				BOP	20	5		
Derks 2022 [33]	RCT	Test group: BG	66	PPD	66	4.9	1.8	Both groups showed improvement in PPD and RBL with no significant differences overall; buccal recession was significantly lower in the test group (*p* = 0.02).
				RBL	66	4.7	1.4	
				BOP	66	47.8		
				REC	66	0.7	1	
				CAL	66	5.6	1.67	
		Control group: OFD	67	PPD	67	4.6	1.8	
				RBL	67	5.2	1	
				BOP	67	44.8		
				REC	67	1.2	1.4	
				CAL	67	5.6	1.67	
Rakasevic 2023 [34]	RCT	Test group: BG	7	PPD	7	2.51	0.9	BG + HA vs. BG showed significantly greater radiographic defect fill.
				RBL	7	0.51	0.9	
				CAL	7	2.5	1.92	
		Test group: HA	6	PPD	6	2.34	0.4	
				RBL	6	0.49	0.7	
				CAL	6	2.34	1.67	
Wang 2023 [35]	Evaluation of RCT	Test group: GBR	12	PPD	12	4.59	0.72	Only GBR arm extracted, The laser group demonstrated greater PPD reduction and bone gain than the control group over follow-up.

## Data Availability

No new data were created or analyzed in this study. Data sharing is not applicable to this article.

## Supplementary Materials

The following supporting information can be downloaded at: https://www.mdpi.com/article/10.3390/dj14030180/s1, Table S1: PRISMA 2020 Checklist; Table S2: Detailed electronic search strategies used for PubMed, Scopus, and Web of Science.

## Abbreviations

The following abbreviations are used in this manuscript:

mBI	Modified Bleeding Index
PPD	Probing Pocket Depth
RBL	Radiographic Bone Level
BOP	Bleeding on Probing
SUP	Suppuration
REC	Mucosal Recession
CAL	Clinical Attachment Level
OFD	Open Flap Debridement
GBR	Guided Bone Regeneration
PRF	Platelet-Rich Fibrin
HA	Hyaluronic Acid
RA	Retrospective analysis
PCS	Prospective Cohort Study
PRISMA	Preferred Reporting Items for Systematic Reviews and Meta-Analyses
RoB2	Cochrane Risk of Bias tool for randomized controlled trials
ROBINS-I	Risk Of Bias In Non-randomized Studies of Interventions
SE	Standard Error
