# Supplementary material for: Regenerative Surgical Treatment of Peri-Implantitis: A Systematic Review and Meta-Analysis
Source: Dent J (Basel). 2026 Mar 18;14(3):180. doi: 10.3390/dj14030180 (PMC13024934; doi:10.3390/dj14030180)
Supplement: Supplementary file 1 [file dentistry-14-00180-s001.zip › Table S2 Detailed electronic search strategies used for PubMed Scopus and Web of Science.pdf]

PubMed search: total : 466

Search 1:

#1 (((Peri-Implantitides[Title/Abstract]) OR (Peri Implantitis[Title/Abstract])) OR (Periimplantitis[Title/Abstract])) OR (Periimplantitides[Title/Abstract])

#1: 4416 result

#2 (guided bone regeneration[Title/Abstract])

#2: 2887 result

#1 AND #2 : 129 result

Search 2:

#1 (((Peri-Implantitides[Title/Abstract]) OR (Peri Implantitis[Title/Abstract])) OR (Periimplantitis[Title/Abstract])) OR (Periimplantitides[Title/Abstract])

#1: 4416 result

#2 2 (hyaluronic acid[Title/Abstract])

#2: 71,254 result

#1 AND #2 : 14 result

Search 3:

#1 (((Peri-Implantitides[Title/Abstract]) OR (Peri Implantitis[Title/Abstract])) OR (Periimplantitis[Title/Abstract])) OR (Periimplantitides[Title/Abstract])

#1: 4416 result

#2 ((platelet rich fibrin[Title/Abstract]) OR (platelet rich plasma[Title/Abstract])

#2: 16953 result

#1 AND #2 : 22 result

Search 4:

#1 (((Peri-Implantitides[Title/Abstract]) OR (Peri Implantitis[Title/Abstract])) OR (Periimplantitis[Title/Abstract])) OR (Periimplantitides[Title/Abstract])

#1: 4416 result

#2 surgical treatment[Title/Abstract]

#2: 179,282 result

#1 AND #2 : 301 result

Scopus search: total: 521

Search 1:

#1 (((Peri-Implantitides[Title/Abstract/key]) OR (Peri Implantitis[Title/Abstract/key])) OR (Periimplantitis[Title/Abstract/key])) OR (Periimplantitides[Title/Abstract/key])

#1: 5442 result

#2 (guided bone regeneration[Title/Abstract/key])

#2: 3735 result

#1 AND #2 : 146 result

Search 2:

#1 (((Peri-Implantitides[Title/Abstract/key]) OR (Peri Implantitis[Title/Abstract/key])) OR (Periimplantitis[Title/Abstract/key])) OR (Periimplantitides[Title/Abstract/key])

#1: 5442 result

#2 (hyaluronic acid[Title/Abstract/key])

#2: 67750

result

#1 AND #2 : 19 result

Search 3:

#1 (((Peri-Implantitides[Title/Abstract/key]) OR (Peri Implantitis[Title/Abstract/key])) OR (Periimplantitis[Title/Abstract/key])) OR (Periimplantitides[Title/Abstract/key])

#1: 5442 result

#2 ({platelet rich fibrin} OR {platelet rich plasma} [Title/Abstract/key])

#2: 5458

result

#1 AND #2 : 12 result

Search 4:

#1 (((Peri-Implantitides[Title/Abstract/key]) OR (Peri Implantitis[Title/Abstract/key])) OR (Periimplantitis[Title/Abstract/key])) OR (Periimplantitides[Title/Abstract/key])

#1: 5442 result

#2 {surgical treatment} [Title/Abstract/key]

#2: 226,333 result

#1 AND #2 : 344 result

Web of science search: total: 1132

Search 1:

#1 (((Peri-Implantitides[ts]) OR (Peri Implantitis[ts])) OR (Periimplantitis[ts])) OR (Periimplantitides[ts])

#1: 5194 result

#2 (guided bone regeneration[ts])

#2: 7330 result

#1 AND #2 : 243 result

Search 2:

#1 (((Peri-Implantitides[ts]) OR (Peri Implantitis[ts])) OR (Periimplantitis[ts])) OR (Periimplantitides[ts])

#1: 5194 result

#2 (hyaluronic acid[ts])

#2: 61.092 result

#1 AND #2 : 12 result

Search 3:

#1 (((Peri-Implantitides[ts]) OR (Peri Implantitis[ts])) OR (Periimplantitis[ts])) OR (Periimplantitides[ts])

#1: 5194 result

#2 ((platelet rich fibrin[ts]) OR (platelet rich plasma[ts])

#2: 24227 result

#1 AND #2 : 39 result

Search 4:

#1 (((Peri-Implantitides[ts]) OR (Peri Implantitis[ts])) OR (Periimplantitis[ts])) OR (Periimplantitides[ts])

#1: 5194 result

#2 (surgical treatment [ts])

#2: 466,875 result

#1 AND #2 : 838 result

Publication range of the studies to be reviewed from 1993 to till 2024

Total number of research added to EndNote: **2119**

Number of duplicate research: **807**

Total number of references to be screened: **1312**

Exclusion after abstract and title screening: **1246**

Number of full version literature not found : **3**

Papers to be fully screened: **63**

Inclusion after full screening**: 12**

The search was conducted on 23/11/2024
